# Detection of Microbial Growth on Indoor Building Materials in Two Countries Using qPCR

**DOI:** 10.3390/microorganisms13071551

**Published:** 2025-07-01

**Authors:** Helena Rintala, Oliver Röhl, Pinja Tegelberg, Teija Meklin

**Affiliations:** 1 Labroc Oy Member of GBA Group, P.O. Box 1199, 70211 Kuopio, Finland; pinja.tegelberg@labroc.fi (P.T.); teija.meklin@labroc.fi (T.M.); 2 GBA Gesellschaft für Bioanalytik mbH, Schelsenweg 24a, 41238 Mönchengladbach, Germany; oliver.roehl@gba-group.de

**Keywords:** indoors, fungi, building material, qPCR, cultivation

## Abstract

According to several reports, 10–50% of buildings in Europe and worldwide suffer from moisture problems, which can lead to microbial growth in building materials. Unrepaired moisture and microbial damage can lead to the degradation of building structures and reduce visual appeal, resulting in economic losses; they can also result in adverse health effects for the building’s occupants. Consequently, robust and reliable methods for the detection of abnormal microbiological conditions in buildings are needed, alongside skilled technical investigations, to plan appropriate renovation actions. In this work, 964 building material samples, which were obtained as part of routine building investigations in two countries, were analyzed for their fungal content using the qPCR method. Cultivation analysis was performed using the same samples, according to corresponding national guidelines. In a sample subset, the total cell counts after staining with acridine orange were determined. The microbial concentrations obtained with all three methods correlated well. Threshold values for the qPCR results were determined using cultivation as a reference method for both countries separately, with similar values obtained for both datasets. Hence, qPCR has great potential to become a standard method of detecting microbes in indoor environments.

## 1. Introduction

Microbes in our living environment impact human health, both beneficially and adversely. We spend most of our time in various indoor environments, the microbial composition of which has a considerable impact on our health. The indoor microbiome consists of microbes from various sources, such as outdoor air, occupants, pets, human activities, building materials, potting soil, etc. Moist building materials are an important source of microbes and a potential cause of adverse health effects. Excess moisture due to leakages, condensation, etc., can lead to microbial growth on building materials, resulting in adverse effects on the respiratory health of the occupants [1]. In addition, moisture and microbial damage can reduce the technical performance of building structures and their visual appeal, which decreases the economic value of the building. According to various reports, 10–50% of buildings across Europe and worldwide suffer from moisture and/or mold problems [2,3,4]. Hence, there is a need to recognize abnormal, potentially harmful microbiomes in our living environments. Dust samples have been used in numerous research projects to study the association of indoor microbiome with health or building metrics [5,6]. The microbial composition of indoor air and dust, which is greatly influenced by outdoor air and the occupants of a building, fluctuates strongly in time and space [7], and shifts in the microbiome caused by microbes from moisture damage may be masked. In routine investigations aiming to detect potential moisture and/or microbial damage in a building, a walkthrough technical inspection and sampling of building materials from suspected or known moisture problem areas allow for a more robust method for assessing the microbial condition of the building, compared to air or dust samples [8].

Methods currently used for the detection of microbes in indoor environments include the morphological identification of cultivated microorganisms, which are collected from the air using impaction or filtration, or the cultivation of swab samples or building materials. These methods are well established and standardized [9,10,11]. In addition, non-cultivated microbial cells and spores collected directly onto microscopic slides or filters can be counted and identified based on their morphology [12,13]. Methods, such as the determination of fungal or bacterial biomass and enzyme activity, have been introduced [14,15]. However, methods based on morphological identification require lengthy training and high expertise from the operators. As such, methods measuring biomass are better suited to research purposes than routine building investigations. Markers used for biomass, such as ergosterol, are dependent on the physiological state of the cell, and the equipment needed for the measurement is expensive.

DNA-based methods such as quantitative PCR (qPCR) are a promising alternative to morphology-based methods in research and routine diagnostics. They are widely used, for example, when detecting pathogens in food or human specimens [16]. DNA-based methodologies were introduced to indoor microbiology at the beginning of the 21st century [17]. One benefit of the DNA-based detection of microbes is that non-cultivable microorganisms are also detected. This allows old, dry microbial damage to be detected if the DNA is not degraded. It has been established that, in any environment, only a small part of microbial diversity can be cultivated [18]. However, studies have shown that dead microbes can also cause adverse health effects [19]. In a recent study, the qPCR-based determination of the total fungal load showed promise in associating respiratory health symptoms with a higher concentration of fungi in dust [20]. One further advantage of the qPCR method is that the analysis time is much shorter than that of cultivation. DNA isolation and qPCR analysis can be performed within a single working day, whereas for cultivation, fungi must be incubated for several days to manifest the morphological characteristics, which is necessary for precise identification. Some species, such as *Penicillium*, require the time- and cost-consuming use of several agar medium types for proper identification. Sometimes, speed is of the essence on a construction site.

Cultivation methods are still the gold standard for examining possible microbial growth on building materials; they are easy to perform, do not require expensive devices, and are, to some extent, standardized. A few countries, for example, Finland and Germany, have even set national guideline values for cultivable microorganisms in buildings [21,22]. Nevertheless, experienced staff are needed for the identification of microbes based on morphological criteria, which makes it nearly impossible to scale up a laboratory in a short amount of time. DNA-based methods, such as qPCR, are being increasingly used in connection with building microbiology; hence, more information is needed about their performance in relation to other methods to interpret the results and better understand the advantages and disadvantages of their use.

The aim of this work was to demonstrate that quantitative real-time PCR produces comparable results with cultivation and total cell count and that qPCR can be used alongside cultivation to detect indoor microbes in routine building investigations.

## 2. Materials and Methods

The first dataset consisted of 630 building material samples sent to a laboratory in Finland to analyze their microbial content. The most common material types were insulation wool (40%), wooden materials (15%), gypsum board (8%), ceramic products (5%), and plastic materials (5%). The samples were collected and analyzed in 2011–2013 in the laboratory using the cultivation method recommended by the Finnish governmental authorities [21]. The samples were weighed (0.5–5 g) and suspended in a 0.9% NaCl solution with Tween 80 (Sigma-Aldrich, Darmstadt, Germany) added to reduce surface tension. NaCl solution was added to a final dilution of 1:10 (*w*/*v*) or 1:100 (*w*/*v*) for the light materials, such as insulation wool. The samples were held in a Sonica 4200 RTH ultrasonic bath (Soltech S.r.l., Milan, Italy) for 30 min and shaken in a MultiReax test tube shaker (Heidolph Scientific Products GmbH, Schwabach, Germany) for one hour to detach the microbes from the material and bring them into the solution. A 10-fold dilution series was prepared, and 0.1 mL of each dilution was spread on 2% malt extract agar (malt extract 20 g/L, agar 15 g/L, chloramphenicol 100 mg/L) (Tammer BioLab Oy, Tampere, Finland) and dichloran–glycerol agar (5 g/L casein digest, 10 g/L glucose, 1 g/L potassium dihydrogen phosphate, 0.5 g/L magnesium sulfate, 0.002 g/L dichloran, 220 g/L glycerol, 13.5 g/L agar, 0.1 g/L chloramphenicol) (Tammer BioLab Oy, Tampere, Finland). Subsequently, they were incubated at 25 °C for 7 days. Fungal colonies were counted and morphologically identified using a light microscope. Then, the concentration of colony-forming units (cfu/g) was calculated in the analyzed material.

The second dataset consisted of 334 building material samples sent to the laboratory in Germany. Styrofoam was the most common material (54%), followed by insulation wool (7%) and ceramic products (7%). The samples were collected and analyzed in 2024. These samples were analyzed in the local laboratory using the cultivation method according to the guidelines published by the German Ministry for Environment [22]. First, 0.1 g of each sample was shredded using scissors or razor blades. The sample was then suspended in 10 mL of a Tween/NaCl buffer (NaCl 8.5 g/L; Tween 80 (Carl Roth GmbH + Co. KG, Karlsruhe, Germany) 0.1 g/L; in distilled water) and shaken on an IKA KS 130 basic horizontal shaker (IKA-Werke GmbH & Co. KG, Staufen, Germany) for 30 min at 300 rpm, according to the guidelines [11]. After that, a 10-fold dilution series was prepared and 0.1 mL of each dilution was spread on malt extract agar (MEA; 12.75 g/L malt extract, 2.75 g/L dextrose, 2.35 g/L glycerol, 0.78 g/L peptone, and 15 g/L agar) (Oxoid Deutschland GmbH, Wesel, Germany) and dichloran–glycerol agar (DG18; 5 g/L peptone, 10 g/L glucose, 1 g/L potassium dihydrogen phosphate, 0.5 g/L magnesium sulfate, 0.002 g/L dichloran, 220 g/L glycerol, 0.05 g/L chloramphenicol, 0.05 g/L chlortetracycline, and 15 g/L agar) (Oxoid Deutschland GmbH, Wesel, Germany). Samples were incubated for 7 to 10 days, and morphologically distinguishable molds were counted and identified.

For the Finnish samples, 1 mL of the microbial suspension obtained for the cultivation method was immediately frozen at −20 °C until DNA isolation. qPCR analyses were performed during 2012 and 2013. For the German samples, an aliquot of 1 mL was sent to Finland for qPCR analysis. The suspensions were cooled to approximately 4–8 °C for shipment and stored in Neopor boxes. Upon arrival, the samples were frozen at −20 °C until DNA isolation.

For DNA isolation, 0.1 mL of the suspension was spiked with salmon sperm DNA (Sigma-Aldrich, St. Louis, MO, USA) as an internal standard and subjected to DNA isolation. For the Finnish dataset, manual DNA extraction was performed using the High Pure PCR Template Preparation Kit (Roche Diagnostics GmbH, Mannheim, Germany). First, we added 0.5 g of glass beads 212–300 µm diameter (Sigma-Aldrich, St. Louis, MO, USA) to the samples; cells were disrupted by 1 min of mechanical bead beating in a homogenizer (Roche Diagnostics GmbH, Mannheim, Germany). Subsequently, the DNA was purified according to the kit’s instructions. For the German dataset, the Fisherbrand Bead mill 24 homogenizer (Fisher Scientific, Loughborough, United Kingdom) was used for cell disruption, and the MagMAX™ Plant DNA Isolation Kit (Applied Biosystems by Thermo Fisher Scientific, Vilnius, Lithuania) was used for DNA isolation with the KingFisher Duo Prime Purification System (Thermo Fisher Scientific, Waltham, MA, USA). Negative (reagents) and positive (fungal mock community) controls were included in the DNA extraction step along with the samples.

The samples were analyzed for their total concentration of fungi using previously published universal fungal qPCR primers [23]. Real-time PCR was conducted with LightCycler 480 (Roche Diagnostics GmbH, Mannheim, Germany) for the Finnish dataset and QuantStudio3 (Applied Biosystems by Thermo Fisher Scientific) for the German dataset. Briefly, qPCR reactions consisted of a mixture of 10 μL LightCycler 480 Probes Master (Roche Diagnostics GmbH, Mannheim, Germany) for the Finnish dataset or TaqMan Fast Advanced Master Mix (Applied Biosystems by Thermo Fisher Scientific, Vilnius, Lithuania) for the German dataset, 2 μL bovine serum albumin (2 mg/mL) (Applied Biosystems by Thermo Fisher Scientific, Vilnius, Lithuania), 1000 nM forward and reverse primers, an 80 nM TaqMan probe, and 2 μL of template DNA filled to a total volume of 20 μL with nuclease-free water (VWR Chemicals). Duplicate reactions were performed in 0.2 mL LightCycler 480 Multiwell Plate 96 (Roche Diagnostics GmbH, Mannheim, Germany) for the Finnish dataset or 0.2 mL Semi-skirted 96-well PCR Plates (Thermo Scientific, Mexico). Non-template controls (NTCs) and positive controls (DNA isolated from a target organism) were included in each run alongside the samples. The number of microbial cell equivalents in the samples was calculated using the previously described relative quantification method [24] and presented as the cell equivalent per gram (CE/g) of building material.

To investigate the correlation between the total cell counts and total fungal DNA, the former was determined for both living and dead cells in a subsample of the German sample set using fluorescent microscopy [25]. Therefore, 1 mL of the suspension, prepared for cultivation and qPCR analyses, was filtered on a Nucleopore membrane filter with 0.2 µm pore size (Macherey-Nagel Vertrieb GmbH & Co. KG; Düren NRW; Germany) using a Laboport N96 vacuum pump (KNF Neuberger GmbH, Freiburg, Germany). Samples were washed with 5 mL of distilled and sterilized water to ensure that the entire number of cells was bound to the filter and nothing remained attached to the filtration vessel. Finally, 100 mg of an acridine orange (Carl Roth GmbH + Co. KG, Karlsruhe, Germany) staining solution was added to the sample and incubated for 24 h. The remaining staining solution was removed via filtration, and the stained filter was stored in a dark and sterile container until microscopic investigation with an Axiolab 5 microscope (Carl Zeiss Microscopy GmbH, Jena, Germany). For fluorescence microscopy, a 445 nm LED Modul with a filter set 70 HE Alexa 430 nm/module (Carl Zeiss Microscopy GmbH, Jena, Germany) was used. Stained cells were counted using fluorescence illumination; unstained cells were also visible using this method but were double-checked via counted with regular light microscopy. Cell concentrations were calculated and given as cells/g of the building material.

Statistical analyses were conducted with the IBM SPSS Statistical Package version 29.0.2.0. Spearman correlation was used to calculate correlations between the concentrations obtained with different methods. The ROC analysis function was used to draw the curves.

## 3. Results

Cultivation and qPCR results were correlated in both datasets; Spearman’s rho was 0.723 and 0.771 for the Finnish and German datasets, respectively. The correlation was statistically significant (*p* < 0.01) for both datasets. The total fungal concentrations determined using the qPCR method were higher than those of the culture method (Table 1).

**Figure 1:**
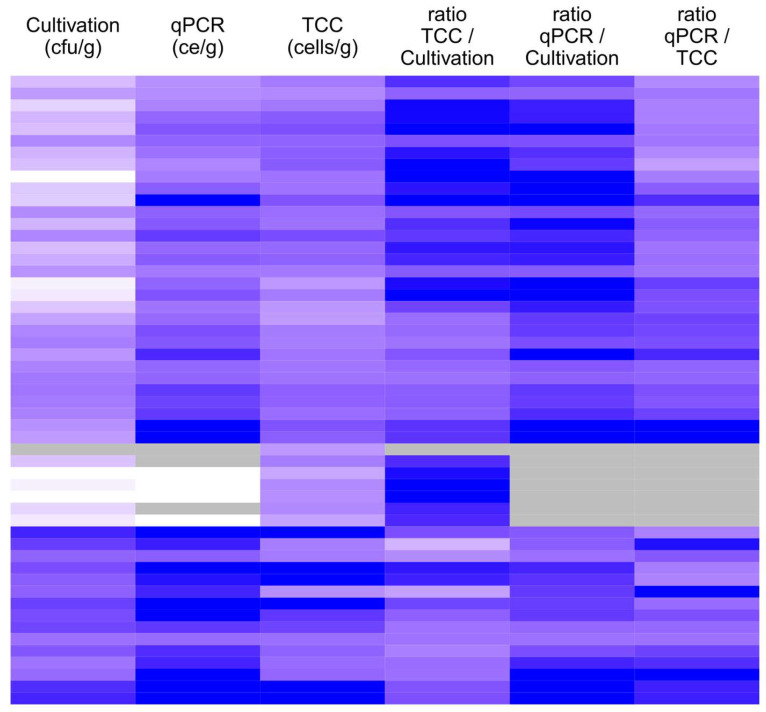
A heatmap showing the total fungal content and ratios of the concentrations determined with three different methods in 53 samples of the German dataset. Each row represents one sample. Rows are clustered with hierarchical clustering with respect to Spearman’s correlation. The first three columns from the left represent concentrations determined using three methods: cultivation, qPCR, and TCC. The color scale from white to blue corresponds to a concentration range from ≤100 to ≥10,000,000. The three columns to the right represent the ratios of concentrations, and the color scale from white to blue corresponds to ratios from ≤0.001 to ≥100. The gray color indicates concentration below the detection or quantification limit or a ratio that could not be calculated as a result.

Altogether, 53 sample suspensions of the German sample set were analyzed for their total cell count (TCC) after treatment with acridine orange. The median concentration of culturable fungi in this subset was 2.2 × 10^4^ cfu/g, whereas the median concentrations determined using qPCR and TCC were 3.5 × 10^5^ ce/g and 1.4 × 10^5^ cells/g, respectively. The minimum concentrations for cultivation and qPCR were below the detection limit and for TCC 8.3 × 10^3^ cells/g. The maximum concentrations for cultivation, qPCR, and TCC analyses were 4.4 × 10^6^ cfu/g, 1.3 × 10^9^ ce/g, and 4.6 × 10^7^ cells/g, respectively. The concentration determined using qPCR and TCC increased with increasing concentration in cultivation. The concentration obtained with qPCR was always higher than the corresponding cultivated concentration. The TCC concentration was lower than the cultivated concentration in four samples, all of which had a concentration higher than 1 × 10^5^ cfu/g (Figure 1). The TCC results were slightly higher in 25% (12/46) of the samples than the qPCR results of the same samples. However, the concentrations obtained with all three methods correlated statistically significantly (*p* < 0.01) with each other (Table 2).

**Figure 2:**
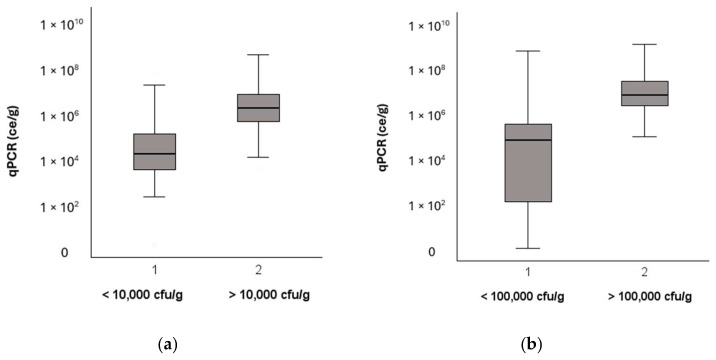
A comparison of cultivation and qPCR results in the Finnish (**a**) and German (**b**) datasets. The samples were divided into two groups based on the guideline value of the cultivation method. Group 1: no microbial growth; Group 2: microbial growth. For the division of groups, the national guidelines were used, which are 10,000 cfu/g for Finland and 100,000 cfu/g for Germany.

Both Finnish and German guidelines for moldy buildings have a threshold value for the total concentration of cultivated fungi, 10,000 cfu/g and 100,000 cfu/g, respectively; samples with values above this are considered to harbor microbial growth. The samples in both datasets were divided into two separate groups: below and above the corresponding guideline value. In both datasets, the total concentration of fungi determined using qPCR was statistically significantly higher (Kruskal–Wallis, *p* < 0.01) in the group of samples that had a cultivated concentration above the guideline value (Figure 2).

**Figure 3:**
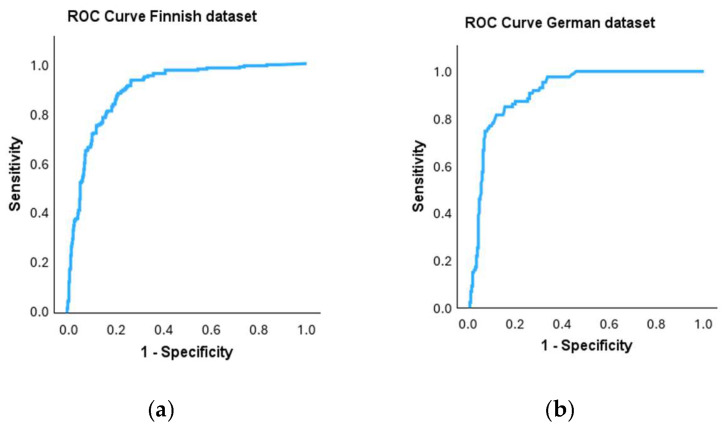
Receiver operating characteristic (ROC) curves of the Finnish (**a**) and German (**b**) data. The curves plot the true positive rate against the false positive rate at each threshold setting of the qPCR concentration using the corresponding cultivation method as the reference.

Receiver operating characteristic (ROC) curves were plotted to investigate the performance of the qPCR method for the binary classification of samples in comparison to the cultivation method, which was used as the reference method (Figure 3a,b). For the ROC curve, the true positive rate is plotted against the false positive rate at each threshold setting. The area under the curve is 0.897 for the Finnish dataset and 0.916 for the German dataset.

The coordinates of the ROC curves were used to determine separate threshold values for the qPCR analysis of both datasets. A threshold value of 100,000 CE/g was determined for the Finnish dataset at the time of analysis. Using that threshold value, 93% of the samples with microbial growth in cultivation were classified similarly based on the qPCR result. In other words, the true positive rate and sensitivity was 93%. Accordingly, 73% of the samples with no microbial growth in cultivation were classified similarly based on qPCR results. Hence, the true negative rate (specificity) was 73% (Table 3(a)). A threshold value of 400,000 CE/g was determined for the German dataset, giving the qPCR method a true positive rate (sensitivity) of 92% and a true negative rate (specificity) of 70% (Table 3(b)).

## 4. Discussion

Microbial concentrations obtained using qPCR and TCC are generally higher than those obtained using cultivation. This may be due to dead and viable but uncultivable microbes in the samples. It has been estimated that even 99% of prokaryotes in environmental samples are uncultivable [26]. Moreover, Hawksworth and Lücking estimated that only around 3–8% of fungal diversity has been discovered so far [27]. Based on the German dataset, 28% of the 272 samples had a cultivation result within one order of magnitude where the corresponding qPCR result was above the limit of quantification. On the other hand, there were 13 samples in which the cultivation result was below the detection limit, with the corresponding qPCR result between 10^4^ and 10^8^ CE/g. Hence, the proportion of uncultivable microbes on building materials varies. This may be due to the age of moisture damage and microbial growth in the material sample; however, more research is needed to prove this. In addition to non-cultivable microbes, samples may contain extracellular DNA released via cell lysis or mediated by predation or phage infection and the release of membrane vesicles [28]. In this work, the qPCR- and TCC-based microbial concentrations of the same sample were mostly within one order of magnitude; in 4 samples out of 53, the total cell count was more than 100 times higher. Hence, the results of this work do not indicate the presence of extracellular DNA as a significant phenomenon. To our knowledge, there are no reports concerning extracellular DNA in building material samples. Most of it would experience degradation during the sample preparation and DNA isolation processes.

In the small subset, in which three methods were compared, the best correlation was observed between qPCR and cultivation results. The total cell count correlated well with the cultivation results but even better with the qPCR results. Differences between cultivation and TCC were most significant at concentrations <10^3^ cfu/g, where the TCC results were constantly 10–1000 times higher than the cultivation results. In concentrations > 10^5^ cfu/g, the TCC result was lower or only slightly higher than the cultivation result. The qPCR/TCC ratio showed an opposite behavior in relation to the qPCR result; the higher the qPCR concentration, the higher the ratio. The results suggest that TCC works better in lower microbial concentrations. The qPCR/cultivation ratio did not show a similar concentration dependency. qPCR has a higher detection limit than the other methods since the sample is diluted multiple times during the analysis. Microbes are ubiquitous, being present at low concentrations everywhere; hence, according to the Finnish and German guidelines, low concentrations are not significant. For the detection of microbial contamination on building materials in relation to moisture damage, the most relevant concentration range is 10^4^–10^6^ cfu/g. All three methods worked in good agreement at the this concentration level.

All methods have disadvantages: cultivation does not detect non-cultivable microbes, fungal cultures grow slowly, and identification requires high-level expertise. However, information about morphologically distinguishable species is obtained. Total cell counting is based on the staining of nucleic acids with acridine orange, and cells that do not contain DNA or RNA and spores with a high melanin content are not stained. Acridine orange is also highly carcinogenic, and the morphological identification of cells and spores requires a lot of time and training. qPCR does not give species information if a larger group is targeted with the assay. However, the qPCR method is quick and works on a wide concentration range. Once the method is set up, performing the analysis does not require high level of expertise. All in all, the results obtained with all three methods correlated well with each other, indicating their ability to detect the microbial contamination of building materials.

Using cultivation as the reference method, a threshold value for the qPCR method was determined for both datasets with the ROC curve and its coordinates. The area under the curve can be used in the evaluation of a diagnostic test. Ideally, the area under the curve should be 1, but generally, figures above 0.8 are considered satisfactory [29]. Using the threshold value, the samples were classified as either harboring or free of microbial growth based on the qPCR result. The threshold values of the cultivation results of the Finnish and German guidelines differed by one order of magnitude. They were independently set based on information collected in research projects in both countries with slightly different setups. The Finnish guidelines were established in the 1990s based on a research project where building materials, which were assessed by trained technical inspectors to be with and without moisture damage, were collected and analyzed. The German guidelines were set in the early 2010s during a research project collecting information about background concentrations of fungi in new and old buildings. The difference in the threshold values may be due to the alternative perspectives used when setting guidelines or the differences in the abundance of microbes because of different climates and building codes. In any case, it is remarkable that the threshold value obtained for the qPCR method in the German dataset was only four times higher than the Finnish dataset. After the Finnish dataset was analyzed, changes were made to the qPCR method, and the threshold value in Finland was re-evaluated and increased to 300,000 CE/g. Therefore, for the qPCR method, the guideline values for both countries are closer together than the cultivation method, indicating that the DNA-based method may be better suited for international standards when determining microbial loads in building materials.

A true positive rate of 93/92% and a true negative rate of 73/70% were achieved for the Finnish/German datasets, respectively. Ideally, the chosen threshold should classify all the samples in the same group as the reference method does, with both the true positive (sensitivity) and true negative rates (specificity) equaling one (or 100%). By lowering the threshold for qPCR, better sensitivity can be achieved (fewer false negatives), and by setting a higher threshold, better specificity is obtained (fewer false positives). Since the cultivation method only detects viable microbes and the qPCR method detects both viable and non-viable microbes, more positive classifications will be achieved with the qPCR method. Bearing this in mind, when setting the threshold value, we strived for good specificity and tried to ensure that all samples classified as positive for fungal growth with cultivation classified similarly in the qPCR analysis. Simultaneously, we accepted the fact that the qPCR method produces “false” positive results. Furthermore, we considered whether the “false” positives of the qPCR method were truly false or if an alternative was true: could the cultivation method produce false negative results? In both datasets, microbial growth was detected in 15%/20% (Finnish/German, respectively) more samples with qPCR than with cultivation. Moisture conditions on building materials can fluctuate greatly over time, and once-wet materials can dry out temporarily or absolutely, causing the microbes living there to lose their vitality. These non-viable microbes still indicate moisture and microbial problems in the building. In addition, the fungi present in moisture-damaged buildings do not lose their harmful features when vitality is lost. Unlike pathogens, they need not be viable to cause adverse health effects. One mechanism behind this may be toxin production. During unfavorable environmental conditions fungi start to produce spores as a survival form, storing toxins in the spores to eliminate competitors in their living environment until the conditions are favorable for growth again [30,31]. Therefore, if the qPCR method has been validated and the threshold value set using cultivation as a reference, the result is valid. Consequently, if the threshold value is exceeded, there is a risk of microbial growth, and hence, the result should be interpreted similarly to when the cultivation threshold value is exceeded.

One difference between the qPCR method and cultivation is that the assay is only designed to detect certain microbes. Thus, it can be designed to detect a single species, a genus, or a larger group, depending on the chosen primers. If a large group of microbes is targeted, such as in this study, the only information gained is the total concentration of all these microbes, not the concentrations of individual species present in the sample. Whether this is important depends on the question at hand. In routine building inspections, it is mostly enough to know that the microbial load in the sample is unusually high. Professionals investigating buildings collect a lot of other information and should be able to interpret the results based on all the data obtained. Most of the building material samples can be classified as contaminated or non-contaminated based on the total concentration of microbes in the sample. In some cases, the occurrence of so-called indicator species or the dominance of one species may provide the investigator with useful information. Cultivation analysis is still needed in occupational settings: it can identify the microbial species that workers have been exposed to. Species information can be important to protect immuno-compromised people, and preserve cultural heritage. In the future, next-generation sequencing methods may provide a useful tool for identifying fungal taxa in indoor environments. NGS techniques have become cheaper and more readily available in recent years and are used in many research projects already [5,20,32,33].

In this work, Finnish and German data were compared for microbial concentrations in building material samples analyzed using the cultivation and qPCR methods. The cultivation analysis was performed, and the samples were categorized according to the guidelines in each corresponding country. Cultivation was used as a reference method to set separate guideline values for the qPCR analysis in both countries. Remarkably similar guideline values were obtained when setting specificity and sensitivity targets to the same level (sensitivity > 90% and specificity > 70%). Recent studies have shown that geographical location and climate factors influence indoor air and dust microbial composition, for example, via varying prevalences and concentrations of fungal taxa in the outdoor air [20,34]. The qPCR detection of total fungi may be an effective method to circumvent the geographical differences in species composition and bring us closer to general guidelines and standardized protocols for detecting microbial growth on building materials.

## 5. Conclusions

qPCR, cultivation, and total cell count all correlated with each other within the relevant concentration range, indicating that all three methods are appropriate for the detection of microbial growth in building materials.

A guideline value for qPCR concentration was determined using cultivation as a reference method, which resulted in good sensitivity and an acceptable specificity, the latter of which is influenced by the fact that qPCR detects both cultivable and non-cultivable microbes.

All methods have their advantages and disadvantages; the advantages of qPCR are its speed and capability to detect both living and dead microbes. Its inability to differentiate between fungal species, if the assay is not species-specific, could be counted as a disadvantage or an advantage.

## Figures and Tables

**Table 1:** The total concentration of fungi in the Finnish and German sample sets determined with culture and qPCR.

	Cultiv. Finland cfu/g (*n* = 630)	qPCR Finland ce/g (*n* = 630)	Cultiv. Germany cfu/g (*n* = 334)	qPCR Germany ce/g (*n* = 334)
Min	<dL ^1^	<dL	<dL	<dL
Median	8.3 × 10^2^	1.1 × 10^5^	8.5 × 10^3^	3.0 × 10^5^
Max	2.8 × 10^7^	6.7 × 10^8^	9.9 × 10^6^	1.4 × 10^9^

^1^ Below the detection limit.

**Table 2:** Correlation coefficients (Spearman’s rho) for a subsample (*n* = 53) of the German sample set.

	Total Cell Count	qPCR
Cultivation	0.574	0.735
qPCR	0.684	1

**Table 3:** The cross-tabulation of classified results for the Finnish (**a**) and German (**b**) samples. Classes are determined based on the interpretation of the cultivation and qPCR results using the respective national guideline values for cultivation and those calculated in this study for the qPCR method.

**(a)**				
	**qPCR**	**No Growth**	**Growth**	**Sum**
**Cultivation**				
No Growth		295 (73%)	108 (27%)	403 (100%)
Growth		15 (7%)	212 (93%)	227 (100%)
Sum		310 (49%)	320 (51%)	630 (100%)
**(b)**				
	**qPCR**	**No Growth**	**Growth**	**Sum**
**Cultivation**				
No Growth		174 (70%)	73 (30%)	247 (100%)
Growth		7 (8%)	80 (92%)	87 (100%)
Sum		181 (54%)	153 (46%)	334 (100%)

## Data Availability

The datasets presented in this article are not readily available due to privacy reasons. Requests to access the datasets should be directed to Teija Meklin or Oliver Röhl.

## Abbreviations

The following abbreviations are used in this manuscript:

CE	Cell equivalent
CFU	Colony-forming unit
NTC	Non-template control
qPCR	Quantitative polymerase chain reaction
TCC	Total cell count
